# Predicting the restrictive eating, exercise, and weight monitoring compulsions of anorexia nervosa

**DOI:** 10.1007/s40519-019-00674-z

**Published:** 2019-03-21

**Authors:** E. Caitlin Lloyd, Maria Øverås, Øyvind Rø, Bas Verplanken, Anne M. Haase

**Affiliations:** 1 Centre for Exercise, Nutrition and Health Sciences, School of Policy Studies, University of Bristol, Bristol, BS8 1TZ UK; 2 Regional Unit for Eating Disorders (RASP), Division of Mental Health and Addiction, Oslo University Hospital, Oslo, Norway; 3 Institute of Clinical Medicine, University of Oslo, Oslo, Norway; 4 University of Bath, Bath, UK

**Keywords:** Anxiety, Anorexia nervosa, Compulsive behaviour, Compulsions

## Abstract

**Purpose:**

Compulsions surrounding restrictive eating, exercise, and weight monitoring are thought to maintain abnormal eating behaviour in individuals with anorexia nervosa (AN). This study aimed to determine if AN psychopathology and trait anxiety explain the presence of restrictive eating, exercise, and weight monitoring compulsions in a mixed sample.

**Methods:**

Participants were 31 females with AN and 31 age and gender-matched healthy individuals (HC). Restrictive eating, exercise and weight monitoring compulsion presence was compared between AN and HC groups. Multivariable poisson regression analyses, adjusted for diagnostic status, were conducted to assess the association of both AN psychopathology and trait anxiety with compulsions across the mixed group.

**Results:**

Individuals with AN endorsed a greater number of restrictive eating, exercise and weight monitoring compulsions compared to HC. In adjusted poisson regression analyses neither AN psychopathology nor trait anxiety predicted compulsion presence: incidence rate ratio (IRR) for AN psychopathology = 1.15 [95% CI 0.84, 1.57], *p* = 0.39; IRR for trait anxiety = 1.01 [95% CI 0.97, 1.06], *p* = 0.50.

**Conclusions:**

Greater presence of restrictive eating, exercise and weight monitoring compulsions was reported by individuals with AN, supporting the conceptualisation of disorder behaviours as compulsive. The study was underpowered to robustly evaluate the association between predictors of interest and the compulsions outcome, largely owing to the small sample size. Further investigation is required, ideally using methods able to identify causal and mediation effects.

**Level of evidence:**

Level V, cross-sectional study.

## Introduction

Anorexia nervosa (AN) has a range of severely detrimental effects on physical wellbeing [1], and the highest mortality rate of any psychiatric condition [2]. These adverse outcomes arise from individuals with AN consistently restricting their intake, such that a significantly low weight is maintained [3].

The inadequate calorie intake of individuals with AN is supported by rituals surrounding restrictive eating, exercise and weight monitoring. At mealtimes individuals with AN tend to eat in very particular ways, for example cutting food into tiny pieces, and completing meals extremely slowly [4]. Engagement in rule-driven and repetitive schedules of exercise [5, 6], and body checking, or stereotyped weight monitoring behaviour [7], is also common to individuals with AN. It is thought that body checking behaviours foster fears and preoccupations with eating and weight-gain, to encourage the continued dietary restriction that is achieved directly by eating and exercise behaviours [8, 9].

The rituals surrounding restrictive eating, exercise and weight monitoring are maladaptive for individuals with AN given their need to gain weight. Individuals with AN report having little control over the rituals, and feeling a “need” to engage in them, despite often simultaneously expressing desires to recover [10]. As such, the rituals endorsed by individuals with AN are suggested to be compulsive [11], compulsivity defined as a trait promoting the persistent repetition of actions that have adverse outcomes [12]. Further, compulsions are well characterised to possess a strong urge-like quality [13]. Given their role in maintaining the low weight of individuals with AN, it would be useful to address compulsive behaviours surrounding restrictive eating, exercise and weight monitoring in AN treatment. For this to be possible; however, the determinants of compulsions must be identified.

Existing literature suggests that AN psychopathology (drive for thinness/restriction, and eating/weight concern [3, 14]) and trait anxiety predict engagement in compulsive behaviour surrounding restrictive eating, exercise and weight monitoring. AN psychopathology is associated with more frequent body checking, and compulsive exercise, in clinical and community samples [15–18]. Using a novel measure of compulsive starvation, Godier and Park [19] found this construct to be positively associated with AN psychopathology in a healthy control (HC) and AN group. The severity of rituals surrounding restrictive eating, exercise and weight monitoring, in terms of the interference with daily functioning and distress caused, also increases with greater AN psychopathology [20].

Trait anxiety is positively associated with restrictive eating behaviour [21] and compulsive exercise [22] in AN populations, and with compulsive exercise in community samples [23]. Furthermore, trait anxiety predicts a greater frequency and duration of episodes characterised by high levels of state anxiety [24], and state anxiety is associated with an increased likelihood of engaging in restrictive eating, exercise, and weight monitoring behaviour in individuals with AN [25–27]. The role of anxiety in behaviour typical of AN is particularly important to clarify, given treatment tends to focus on weight-restoration and addressing eating disorder specific cognition, as opposed to more general psychopathology.

The current study aims to confirm the presence of compulsions surrounding restrictive eating, exercise and weight in AN. In addition, the study will evaluate the relationships of AN psychopathology and trait anxiety with compulsive behaviour typical of AN in a clinical and community population, with a view to informing how AN behavior may be maintained. It was hypothesised that greater levels of AN psychopathology, and greater levels of anxiety, would be associated with greater engagement in compulsions typical of AN – in AN and HC groups.

## Methods

### Data sources

Data for the present study was originally collected to investigate memory and perception of body image in AN [28]. The study was completed at the Regional Department for Eating Disorders (RASP), Oslo, Norway, and approved by the Regional Ethical Committee for Medical Research. Informed written consent was obtained from all participants, or from parents of participants when participants were under the age of 16 (the legal age at which consent may be provided in Norway).

### Participants

Fifty females with AN were included in the original study, recruited from 5 specialist eating disorder units in Norway (inpatient and outpatient). Once AN participant recruitment was complete, 35 healthy adolescent/young adult females (HC) from schools and universities local to RASP were recruited. This group was selected on the basis of having a similar age-distribution to the AN sample.

Participants were excluded from the current investigation if they were missing measures of the study variables. Of the originally recruited participants, 13 individuals (11 AN and 2 HC) had not completed the measure assessing the presence of eating, exercise and weight monitoring compulsions, and one AN participant was missing trait anxiety information.

The AN group were administered the Eating Disorder Examination, based on the Diagnostic and Statistical Manual of Mental Disorders, fourth edition (DSM-IV [29]), to validate diagnoses. Duration of illness was assessed by way of self-report questionnaire. Height and weight of AN participants was assessed by clinical staff and reported to researchers if participants consented, for calculation of body mass index (BMI). Seven of the recruited AN group were excluded from the current investigation because records indicated that they did not meet any AN criteria (*n* = 3), or did not experience psychopathology that is typical of AN (*n* = 4). The Norwegian translation of the Eating Disorders Examination self-report, the EDE-Q [30], was used to screen HC for AN psychopathology. Three HC were excluded because their global EDE-Q scores were above four, thus indicating clinically significant AN psychopathology. The EDE-Q is described further in the measures section. Researchers recorded the BMI of HC during the study assessment, following height and weight measurement.

Participants of the current study comprised 31 AN, and 31 HC, who were aged between 14 and 27. Of the AN group, 20 participants met all DSM-IV criteria for AN. Eleven individuals with AN had a bodyweight that was higher than 85% of that expected, due to weight increases resulting from hospital treatment, and/or did not meet menstruation criteria. These participants were included. Amenorrhea is no longer a criterion for AN [3], and we were interested in the factors underlying engagement in maladaptive behaviour that can persist following weight-gain. There were no significant differences in trait anxiety, AN psychopathology, and endorsement of eating, exercise and weight-related compulsions, between individuals meeting full versus partial criteria for AN.

### Measures

#### Explanatory variables

Trait anxiety was measured by the trait anxiety subscale of the State-Trait Anxiety Inventory (STAI; [31]). AN psychopathology was indexed by global score on the Norwegian translation of the EDE-Q [30]. The EDE-Q assesses AN psychopathology: desire for thinness and weight-loss; fears surrounding eating and weight-gain; and dissatisfaction and preoccupation with weight, shape and eating. The global score is the average of four subscales: restraint; weight concern; shape concern; and eating concern. Both the EDE-Q and STAI are well established to have excellent psychometric properties and are commonly used to assess the constructs of interest [32–37]. The Norwegian translation of the EDE-Q has demonstrated satisfactory psychometric properties [38].

#### Outcome variable

The presence of eating, exercise and weight monitoring-related compulsions was assessed by the compulsions severity subscale of the Child Obsessive Compulsive Inventory (ChOCI; [39]). In particular, we were interested in responses to the question that asks participants to name their 3 most severe, or upsetting, compulsions. Responses to this question indicated the extent to which participants experience severe compulsions related to eating, exercise or weight monitoring. Participants are guided that a compulsion is something they feel they ‘have to do and cannot stop’ to ensure relevant behaviours are reported. The ChOCI is reported to reliably and validly measure obsessive and compulsive symptom severity in adolescents [39], and so was deemed appropriate for use in a mid-adolescent/early adulthood sample.

An independent rater determined whether named compulsions were related to restrictive eating, exercise or weight monitoring. The number of such compulsions was recorded, to comprise the count variable ‘restrictive eating, exercise and weight monitoring compulsions’, which had possible values of 0 to 3. The presence of restrictive eating, exercise and weight monitoring compulsions is a marker of abnormal behaviour, as opposed to cognition, surrounding eating, exercise, and weight.

## Data preparation and analysis

All analyses were conducted using the statistics program Stata [40]. Mean AN psychopathology and trait anxiety, of AN and HC, was compared using *t* tests. The number of restrictive eating, exercise and weight monitoring compulsions of AN and HC groups was compared using a Chi-square test. The correlation between AN psychopathology and trait anxiety, both unadjusted and adjusted for diagnostic status, was calculated.

Univariable (single predictor) poisson regression models estimated the association of the predictors AN psychopathology and trait anxiety with the compulsions count variable. A multivariable poisson regression model, adjusted for diagnostic status (i.e., AN versus HC), assessed the independent association of each predictor variable with the compulsions outcome. Model coefficients indicate the increased log of expected compulsions count per one unit increase in the predictor. The coefficients were exponentiated to produce incidence rate ratios, indicating the increase in expected compulsions count per one unit increase in the given predictor.

Exploratory analyses assessed whether there was a difference between AN and HC groups in the association of predictors (trait anxiety and eating disorder psychopathology) with restrictive eating, exercise and weight monitoring compulsions. This was achieved by adding relevant interaction terms to the poisson regression model. The sample size was such that interaction effect coefficients could not be estimated with confidence, and outcomes of this analysis are not reported; further details are available upon request.

## Results

### Sample characteristics

Table 1 details the demographic and clinical characteristics of the sample. AN psychopathology was much higher in AN as compared to HC (Cohen’s *d* for between group differences = 3.00), as was trait anxiety (Cohen’s d for between group differences = 3.22). Individuals with AN also reported a greater number of compulsions compared to HC.

**Table 1:** Participant characteristics

	Women with AN (*N* = 31)	Healthy women (*N* = 31)	*T* statistic (*p*)
	*M* (SD)	*M* (SD)	
Age^a^	19.6 (3.27)	18.6 (3.71)	1.10 (0.28)
BMI (kg/m^2^)^b^	16.33 (2.13)	21.78 (2.73)	8.58 (< 0.001)
Duration of illness (months)	32 (27.55)		
AN psychopathology	3.62 (1.09)	0.81 (0.75)	11.87 (< 0.001)
Trait anxiety	62.81 (7.23)	37.10 (8.68)	12.68 (< 0.001)

	Count (*N*)	Count (*N*)	*χ*^2^ statistic (*p*)
Number of eating, exercise and weight monitoring compulsions			
0	8	26	22.60 (< 0.001)
1	10	4	
2	7	1	
3	6	0	

^a^Precise age information was missing for one HC

^b^BMI data for one AN participant, and two HC, was missing

### Preliminary analyses

AN psychopathology and trait anxiety were correlated (*R* = 0.82, *p* < 0.001). This association remained but was much weaker when adjusting for diagnostic status (*R* = 0.20, *p* = 0.12). Greater AN psychopathology and trait anxiety were associated with an increased rate of eating, exercise and weight monitoring compulsions in univariable models (Table 2).

**Table 2:** Univariable poisson regression models for the prediction of restrictive eating, exercise and weight monitoring compulsions by trait anxiety and AN psychopathology

Univariable regression of compulsions		Coefficient estimates				Model statistics	
Explanatory variable		*B*	SE	Incident rate ratio [95% CI]	*p* value	*χ*^2^ (*df*)	Pseudo *R*^2^
Model 1	AN psychopathology	0.46	0.10	1.59 [1.31, 1.93]	< 0.001	25.96 (1) *p* < 0.001	0.17
	Constant	− 1.57	0.37	0.21 [0.10, 0.42]	< 0.001		
Model 2	Trait anxiety	0.06	0.01	1.06 [1.03,1.08]	< 0.001	26.52 (1) *p* < 0.001	0.17
	Constant	− 3.35	0.74	0.03 [0.01, 0.15]	< 0.001		

### Main analyses

The multivariable regression model accounted for 21.0% of the variance in the count of restrictive eating, exercise and weight monitoring compulsions; *χ*^2^(3) = 32.18, *p* ≤ 0.001. There was no strong evidence to support any of the predictors explaining unique variance in the compulsions outcome. Table 3 provides further information.

**Table 3:** Adjusted Multivariable poisson regression model for the prediction of restrictive eating, exercise and weight monitoring compulsions by AN psychopathology and trait anxiety

Multivariable regression of compulsions	Coefficient estimates				Model statistics	
Explanatory variable	*B*	SE	Incident rate ratio [95% CI]	*P* value	*χ*^2^ (*df*)	Pseudo *R*^2^
Diagnostic status (AN)	1.19	0.73	3.30 [0.80, 13.68]	0.10	32.18 (3) *p* ≤ 0.001	0.21
AN psychopathology	0.14	0.16	1.15 [0.84, 1.57]	0.39		
Trait anxiety	0.01	0.02	1.01 [0.97, 1.06]	0.50		
Constant	− 2.30	0.88	0.10 [0.02, 0.56]	< 0.01		

## Discussion

This study found that individuals with AN endorsed a greater number of restrictive eating, exercise and weight monitoring compulsions compared to HC, supporting behaviours typical of AN being conceptualised as compulsive [4]. The compulsive nature of disorder behaviours likely promotes the persistence of AN, and thus it is important to understand the determinants of disorder-relevant compulsions in order to develop effective treatment interventions.

The present study particularly sought to understand whether AN psychopathology and trait anxiety predicted compulsive behaviours centred on restrictive eating, exercise and weight monitoring, in individuals with AN and HC. In univariable models, greater AN psychopathology and greater trait anxiety predicted an increased number of AN compulsions. The direction of association was consistent in multivariable models adjusted for diagnostic status, however, the strength of association was reduced and point estimates less precise (for both trait anxiety and AN psychopathology). Neither AN psychopathology nor trait anxiety was able to explain unique variation in compulsion presence beyond that accounted for by diagnostic status. This contrasts with findings of other studies. Previously relationships between AN psychopathology/anxiety and compulsive behaviour surrounding restrictive eating, exercise and weight monitoring have been reported in populations of either AN or HC individuals [19, 20, 23, 41–43]. One plausible cause of the discrepancy is low power in the current study, mainly owing to a relatively small number of participants—a limitation of the present investigation.

The nature of the compulsions outcome variable will also have limited sensitivity to detect an association between this and AN psychopathology/trait anxiety. Assessment of the number of severe compulsions surrounding restrictive eating, exercise and weight monitoring allowed for the capture of different compulsive behaviours typical of AN. This approach promoted validity in the assessment of whether compulsions were present. However, the range of possible responses was limited. Most individuals with AN reported a non-zero number of compulsions, while most HC reported no compulsions, meaning variability in the response was particularly low within diagnostic status categories—which, like sample size, has implications for statistical power. Individual differences would be captured to a finer degree by alternative measures of compulsivity. Future studies might use multi-item scales that separately assess compulsive starvation, compulsive exercise and body checking. Alternatively, studies might investigate the presence and severity of a variety of specific restrictive eating, exercise and weight monitoring compulsions characteristic of AN using the Yale–Brown–Cornell Eating Disorder Scale [44].

The study has some important strengths, such as the use of strict inclusion criteria to ensure the AN sample was typical of this population, and appropriate modelling of the count outcome variable within poisson regression models. However, given the discussed methodological shortcomings relating to outcome measurement and sample size, we encourage further investigation into predictors of restrictive eating, exercise and weight monitoring compulsions—in AN and HC. In addition to AN psychopathology and trait anxiety, other potentially relevant factors (e.g., cognitive processing style, psychiatric comorbidity) may be studied for their association with compulsive behaviour typical of AN. Ideally studies would be adequately powered to explore interaction effects, to understand how predictors of compulsive behaviour may differ between AN and HC. To gain further insight, it would be useful to compare AN subtypes for severity of various illness-related compulsions, as well as for predictors of these compulsions. Future studies might also examine the mechanisms by which determinants are associated with compulsive behaviour surrounding restrictive eating, exercise and weight monitoring, to identify factors that might most usefully be targeted in AN treatment. For example, in this study anxiety and AN psychopathology were related, consistent with findings from studies in clinical [45, 46] and subclinical [47] populations. Should, as has been proposed (e.g., [48–50]), anxiety exert a causal influence on AN psychopathology, and should AN psychopathology in turn cause compulsive behaviour typical of AN, it might be advantageous to address general anxiety (i.e., not that specific to eating and weight-gain) for reduction of both cognitive and behavioural symptoms of AN. Cross-sectional studies such as the present one cannot make inferences regarding the direction of observed associations. For rigorous tests of causal and mechanistic hypotheses longitudinal and experimental designs should be implemented.

## Conclusion

Our findings support AN being characterised as a compulsive disorder, highlighting the importance of identifying determinants of compulsive behaviour surrounding restrictive eating, exercise and weight monitoring to inform AN treatment. The study did not find strong evidence to support trait anxiety or AN psychopathology being associated with greater engagement in compulsive behaviour typical of AN, in a clinical or community population. The direction of the associations that were observed was, however, consistent with findings of previous studies that do support such relationships to exist. The present investigation was underpowered to assess associations robustly, and further investigation is encouraged—particularly using designs able to establish causality and elucidate mechanisms underpinning causal effects.

## References

[1] Mehler P, Brown C (2015). Anorexia nervosa–medical complications. J Eat Disord.

[2] Steinhausen H-C (2002). The outcome of anorexia nervosa in the 20th century. Am J Psychiatry.

[3] American Psychiatric Association (2013). Diagnostic and statistical manual of mental disorders (DSM-5^®^).

[4] Gianini L, Liu Y, Wang Y, Attia E, Walsh BT, Steinglass J (2015). Abnormal eating behavior in video-recorded meals in anorexia nervosa. Eat Behav.

[5] Fietz M, Touyz S, Hay P (2014). A risk profile of compulsive exercise in adolescents with an eating disorder: a systematic review. Adv Eat Disord Theory Res Pract.

[6] Young S, Touyz S, Meyer C, Arcelus J, Rhodes P, Madden S (2017). Validity of exercise measures in adults with anorexia nervosa: the EDE, compulsive exercise test and other self-report scales. Int J Eat Disord.

[7] Kraus N, Lindenberg J, Zeeck A, Kosfelder J, Vocks S (2015). Immediate effects of body checking behaviour on negative and positive emotions in women with eating disorders: an ecological momentary assessment approach. Eur Eat Disord Rev.

[8] Bailey N, Waller G (2017). Body checking in non-clinical women: Experimental evidence of a specific impact on fear of uncontrollable weight gain. Int J Eat Disord.

[9] Calugi S, El Ghoch M, Dalle Grave R (2017). Body checking behaviors in anorexia nervosa. Int J Eat Disord.

[10] Godier LR, Park RJ (2015). Does compulsive behavior in Anorexia Nervosa resemble an addiction? A qualitative investigation. Front Psychol.

[11] Godier LR, Park RJ (2014). Compulsivity in anorexia nervosa: a transdiagnostic concept. Front Psychol.

[12] Robbins TW, Gillan CM, Smith DG, de Wit S, Ersche KD (2012). Neurocognitive endophenotypes of impulsivity and compulsivity: towards dimensional psychiatry. Trends Cogn Sci.

[13] Gillan CM, Robbins TW (2014). Goal-directed learning and obsessive–compulsive disorder. Phil Trans R Soc B.

[14] Cooper Z, Fairburn C (1987). The eating disorder examination: a semi-structured interview for the assessment of the specific psychopathology of eating disorders. Int J Eat Disord.

[15] Homan K (2010). Athletic-ideal and thin-ideal internalization as prospective predictors of body dissatisfaction, dieting, and compulsive exercise. Body Image.

[16] Lavender JM, Wonderlich SA, Crosby RD, Engel SG, Mitchell JE, Crow S (2013). A naturalistic examination of body checking and dietary restriction in women with anorexia nervosa. Behav Res Ther.

[17] Stefano EC, Hudson DL, Whisenhunt BL, Buchanan EM, Latner JD (2016). Examination of body checking, body image dissatisfaction, and negative affect using Ecological momentary assessment. Eat Behav.

[18] Noetel M, Miskovic-Wheatley J, Crosby RD, Hay P, Madden S, Touyz S (2016). A clinical profile of compulsive exercise in adolescent inpatients with anorexia nervosa. J Eat Disord.

[19] Godier LR, Park RJ (2015). A novel measure of compulsive food restriction in anorexia nervosa: validation of the self-starvation scale (SS). Eat Behav.

[20] Jordan J, Joyce PR, Carter FA, McIntosh VV, Luty SE, McKenzie JM (2009). The Yale–Brown–Cornell eating disorder scale in women with anorexia nervosa: what is it measuring?. Int J Eat Disord.

[21] Steinglass JE, Sysko R, Mayer L, Berner LA, Schebendach J, Wang YJ (2010). Pre-meal anxiety and food intake in anorexia nervosa. Appetite.

[22] Peñas-Lledó E, Vaz Leal FJ, Waller G (2002). Excessive exercise in anorexia nervosa and bulimia nervosa: relation to eating characteristics and general psychopathology. Int J Eat Disord.

[23] Weinstein A, Maayan G, Weinstein Y (2015). A study on the relationship between compulsive exercise, depression and anxiety. J Behav Addict.

[24] Edmondson D, Shaffer JA, Chaplin WF, Burg MM, Stone AA, Schwartz JE (2013). Trait anxiety and trait anger measured by ecological momentary assessment and their correspondence with traditional trait questionnaires. J Res Pers.

[25] Engel SG, Wonderlich SA, Crosby RD, Wright TL, Mitchell JE, Crow SJ (2005). A study of patients with anorexia nervosa using ecologic momentary assessment. Int J Eat Disord.

[26] Lavender JM, De Young KP, Wonderlich SA, Crosby RD, Engel SG, Mitchell JE (2013). Daily patterns of anxiety in anorexia nervosa: associations with eating disorder behaviors in the natural environment. J Abnorm Psychol.

[27] Meyer C, Taranis L, Goodwin H, Haycraft E (2011). Compulsive exercise and eating disorders. Eur Eat Disord Rev.

[28] Øverås M, Kapstad H, Brunborg C, Landrø NI, Lask B (2014). Memory versus perception of body size in patients with anorexia nervosa and healthy controls. Eur Eat Disord Rev.

[29] American Psychiatric Association (2000). Diagnostic and statistical manual of mental disorders (DSM-IV-TR).

[30] Fairburn CG, Beglin SJ (1994). Assessment of eating disorders: Interview or self-report questionnaire?. Int J Eat Disord.

[31] Spielberger CD, Gorsuch RL, Lushene RE (1970). Manual for the state-trait anxiety inventory.

[32] Berg KC, Peterson CB, Frazier P, Crow SJ (2012). Psychometric evaluation of the eating disorder examination and eating disorder examination-questionnaire: a systematic review of the literature. Int J Eat Disord.

[33] McDowell I (2006). Measuring health: a guide to rating scales and questionnaires.

[34] Mond JM, Hay PJ, Rodgers B, Owen C (2006). Eating Disorder Examination Questionnaire (EDE-Q): norms for young adult women. Behav Res Ther.

[35] Nixon GF, Steffeck JC (1977). Reliability of the state-trait anxiety inventory. Psychol Rep.

[36] Spielberger CD, Gorsuch RL, Lushene R, Vagg PR, Jacobs GA (1983). Manual for the state-trait anxiety inventory.

[37] Vigneau F, Cormier S (2008). The factor structure of the State-Trait Anxiety Inventory: an alternative view. J Pers Assess.

[38] Rø Ø, Reas DL, Lask B (2010). Norms for the Eating Disorder Examination Questionnaire among female university students in Norway. Nord J Psychiatry.

[39] Shafran R, Frampton I, Heyman I, Reynolds M, Teachman B, Rachman S (2003). The preliminary development of a new self-report measure for OCD in young people. J Adolesc.

[40] StataCorp (2015). Stata statistical software.

[41] Haase AM, Mountford V, Waller G (2011). Associations between body checking and disordered eating behaviors in nonclinical women. Int J Eat Disord.

[42] Tasca GA, Balfour L (2014). Attachment and eating disorders: a review of current research. Int J Eat Disord.

[43] Tasca GA, Illing V, Balfour L, Krysanski V, Demidenko N, Nowakowski J (2009). Psychometric properties of self-monitoring of eating disorder urges among treatment seeking women: Ecological momentary assessment using a daily diary method. Eat Behav.

[44] Mazure CM, Halmi KA, Sunday SR, Romano SJ, Einhorn AM (1994). The Yale–Brown–Cornell eating disorder scale: development, use, reliability and validity. J Psychiatr Res.

[45] Spindler A, Milos G (2007). Links between eating disorder symptom severity and psychiatric comorbidity. Eat Behav.

[46] Sternheim L, Startup H, Schmidt U (2015). Anxiety-related processes in anorexia nervosa and their relation to eating disorder pathology, depression and anxiety. Adv Eat Disord.

[47] Davis KR, Fischer S (2013). The influence of trait anger, trait anxiety and negative urgency on disordered eating. Pers Individ Differ.

[48] Kaye WH, Fudge JL, Paulus M (2009). New insights into symptoms and neurocircuit function of anorexia nervosa. Nat Rev Neurosci.

[49] Lloyd EC, Frampton I, Verplanken B, Haase AM (2017). How extreme dieting becomes compulsive: a novel hypothesis for the role of anxiety in the development and maintenance of anorexia nervosa. Med Hypotheses.

[50] Nunn K, Frampton I, Lask B (2012). Anorexia nervosa—a noradrenergic dysregulation hypothesis. Med Hypotheses.

